# Increased *SORBS3* expression in brain ageing contributes to autophagic decline via YAP1-WWTR1/TAZ signaling

**DOI:** 10.1080/15548627.2022.2100106

**Published:** 2022-07-12

**Authors:** So Jung Park, Rebecca A. Frake, David C. Rubinsztein

**Affiliations:** a Department of Medical Genetics, Cambridge Institute for Medical Research, Wellcome Trust/MRC Building, Cambridge Biomedical Campus, Cambridge, UK; b UK Dementia Research Institute, Cambridge Biomedical Campus, Cambridge Biomedical Campus, Cambridge, UK; c Oxford University Hospitals NHS Foundation Trust, John Radcliffe Hospital, Headley Way, Oxford, UK

**Keywords:** Autophagy, brain aging, SORBS3, vinexin, YAP1-WWTR1/TAZ

## Abstract

Impaired autophagosome formation and reduced flux through the macroautophagy/autophagy pathway occurs outside the brain as part of normal aging in various species. We recently identified autophagic decline in mouse brain tissue dependent on aging. This sits alongside significantly increased expression of the *Sorbs3/SORBS3/vinexin* (sorbin and SH3 domain containing 3) gene in older mouse and human brains. We found that SORBS3 negatively regulates autophagy in several cell lines, including mouse primary neurons. SORBS3 depletion increases F-actin structures, which compete with YAP1-WWTR1/TAZ to bind AMOT (angiomotin) proteins in the cytosol. Unbound YAP1-WWTR1/TAZ is free to move into the nucleus and upregulate YAP1-WWTR1/TAZ target gene expression. This upregulates autophagosome formation, in part through increased expression of myosin- and actin-related genes. Moreover, we have shown these YAP1-WWTR1/TAZ target genes are downregulated in older mouse and human brains. Taken together, our findings suggest that increased *SORBS3* expression contributes to autophagic decline in normal brain aging across species.

Autophagy is an intracellular degradation pathway that is fundamental to maintaining cellular homeostasis. Cytoplasmic cargos are sequestered in phagophores that mature into double-membraned autophagosomes, which fuse with lysosomes to remove autophagic substrates. Autophagic degradation serves several important biological functions in the cell, including removing aggregated proteins and damaged or excess organelles, as well as protein quality control. Dysfunctional autophagy has been identified in various normal physiological processes and diseases. Specifically, impaired autophagy is implicated in both normal brain aging and age-related neurodegenerative disease. Autophagic decline is suggested to contribute to an age-related decline in “cellular fitness” through mechanisms such as dysfunctional proteostasis, genomic instability stemming from crosstalk between autophagy and DNA repair systems and impaired clearance of damaged mitochondria causing oxidative stress. Autophagy upregulation is therefore a potential therapeutic strategy in various age-related diseases, including neurodegenerative conditions.

Upregulating autophagy using both pharmacological and genetic approaches prolongs several species’ healthy lifespan and ameliorates phenotypes associated with normal aging. Age-related reductions in expression of the core autophagy genes *ATG5, ATG7* and *BECN1* have been observed in human brain tissue. In addition, several core autophagy proteins exhibit an age-dependent decline in expression in mouse hypothalamus tissue. The decline in autophagy as part of normal brain aging that can be inferred from these studies is especially notable given neurons are post-mitotic cells, unable to segregate dysfunctional proteins and organelles from daughter cells during cell division, and consequently more dependent on autophagy to maintain cellular homeostasis. Despite this, no previously published study has directly examined autophagy flux in aged mammalian brain tissue.

We addressed this gap in the literature in our recent study, which demonstrates autophagic vesicles (autophagosomes and autolysosomes) are significantly reduced in motor cortex from aged transgenic autophagy reporter mice expressing mRFP-GFP-LC3 [1]. In order to identify potential autophagy regulators with relevance to aging, we used RNA sequencing data to examine changes in mRNA expression with chronological age in mouse brain tissue (combined motor cerebral cortex, somatosensory cerebral cortex and striatum), human cerebral cortex and human hippocampus. Interestingly, we found *Sorbs3/SORBS3* mRNA levels are significantly increased in older mouse and human brains. Using siRNA and shRNA gene knockdown techniques, we demonstrated the actin cytoskeleton modulator SORBS3 negatively regulates autophagy in various cell lines, including mouse primary neurons. Our data indicate SORBS3 knockdown promotes autophagy biogenesis, thereby increasing degradation of disease-relevant autophagy substrates, such as mutant HTT (huntingtin) and mutant SNCA^A53T^ (synuclein alpha).

There is increasing evidence to suggest activity of the transcriptional coactivators YAP1 and WWTR1/TAZ is modulated by actin cytoskeleton dynamics. Our laboratory has also recently linked YAP1-WWTR1/TAZ transcriptional activity to autophagosome biogenesis. We therefore examined whether SORBS3 depletion upregulates autophagy via YAP1-WWTR1/TAZ signaling. We found that SORBS3 knockdown increases YAP1-WWTR1/TAZ activity and that autophagy upregulation following SORBS3 depletion is YAP1-WWTR1/TAZ dependent. We went on to characterize a molecular mechanism whereby SORBS3 depletion increases filamentous actin (F-actin) structures, which compete with YAP1-WWTR1/TAZ for binding to the AMOT family of proteins in the cytosol. More frequent binding between F-actin structures and AMOTs therefore releases YAP1-WWTR1/TAZ to enter the nucleus to increase YAP1-WWTR1/TAZ transcriptional activity through TEAD transcription factors (Figure 1).

**Figure 1.**
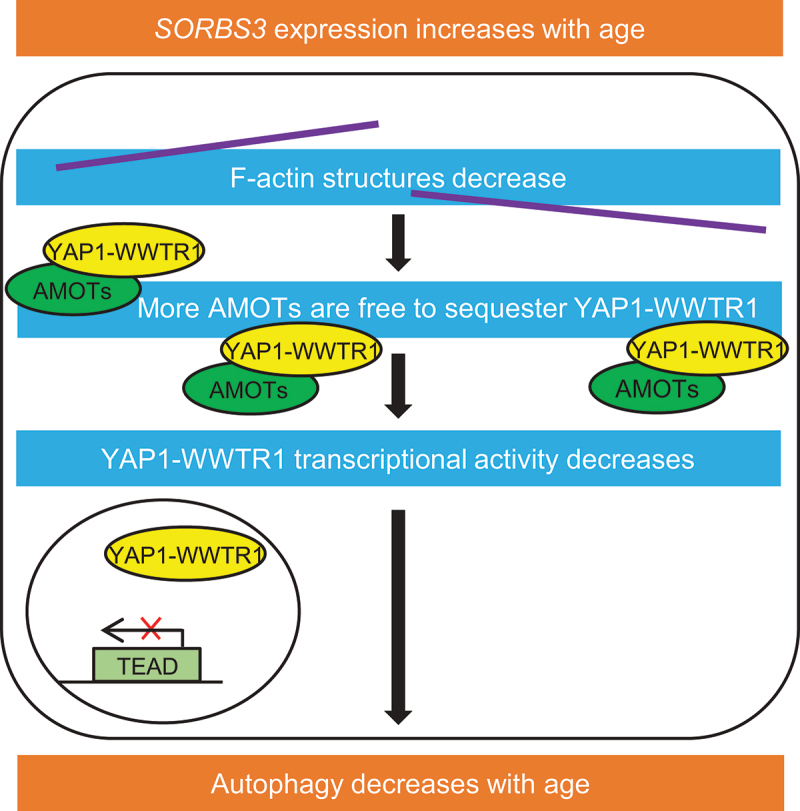
Proposed mechanism for increased SORBS3 expression contributing to autophagic decline in brain aging. *Sorbs3/SORBS3* mRNA expression increases with chronological age in mouse and human brain tissue. This decreases F-actin structures (shown as purple lines). As F-actin and YAP1-WWTR1/TAZ (shown as yellow circles) compete for binding to AMOTs (shown as green circles), the decrease in F-actin structures increases cytosolic YAP1-WWTR1/TAZ-AMOT binding. This reduces YAP1-WWTR1/TAZ target gene expression, which downregulates autophagy.

Our group has recently demonstrated YAP1-WWTR1/TAZ transcriptional activity upregulates autophagy through increased transcription of myosin- and actin-related YAP1-WWTR1/TAZ target genes. Specifically, *MYL9/MLC2* (myosin light chain 9), *MYH10* (myosin heavy chain 10) and several other myosin- and actin-related genes (*MYH9, MYH14, ACTN1* and *ACTB*) participate in autophagosome biogenesis downstream of YAP1-WWTR1/TAZ. We therefore examined the relationship between SORBS3-regulated autophagy and these YAP1-WWTR1/TAZ target genes. We found *MYL9/MLC2, MYH10, MYH9, MYH14, ACTN1* and *ACTB* are all upregulated at the mRNA level upon SORBS3 depletion. Most interestingly, we also identified an age-dependent decrease in *MYL9/Myl2/MLC2* and *Myh10/MYH10* mRNA expression in mouse brain tissue, human cerebral cortex and human hippocampus, which is negatively correlated with increased *Sorbs3/SORBS3* mRNA expression. Taken as a whole, our study characterizes increased SORBS3 expression as a contributor to autophagic decline in normal brain aging via reduced autophagosome biogenesis regulated by YAP1-WWTR1/TAZ transcriptional activity.
